# A virtual biopsy study of microsatellite instability in gastric cancer based on deep learning radiomics

**DOI:** 10.1186/s13244-023-01438-1

**Published:** 2023-06-07

**Authors:** Zinian Jiang, Wentao Xie, Xiaoming Zhou, Wenjun Pan, Sheng Jiang, Xianxiang Zhang, Maoshen Zhang, Zhenqi Zhang, Yun Lu, Dongsheng Wang

**Affiliations:** 1 Qingdao Medical College, Qingdao University, Qingdao, Shandong China; 2 Department of Gastrointestinal Surgery, The Affiliated Hospital of Qingdao University, No. 1677, Wutaishan Road, Qingdao, 266000 Shandong China; 3 Department of Radiology, The Affiliated Hospital of Qingdao University, Qingdao, Shandong China; 4 Department of Pathology, The Affiliated Hospital of Qingdao University, Qingdao, Shandong China; 5 Shandong Key Laboratory of Digital Medicine and Computer Assisted Surgery, Qingdao, Shandong China

**Keywords:** Gastric cancer, Microsatellite instability (MSI), Radiomics, Deep learning, Computed tomography

## Abstract

**Objectives:**

This study aims to develop and validate a virtual biopsy model to predict microsatellite instability (MSI) status in preoperative gastric cancer (GC) patients based on clinical information and the radiomics of deep learning algorithms.

**Methods:**

A total of 223 GC patients with MSI status detected by postoperative immunohistochemical staining (IHC) were retrospectively recruited and randomly assigned to the training (*n* = 167) and testing (*n* = 56) sets in a 3:1 ratio. In the training set, 982 high-throughput radiomic features were extracted from preoperative abdominal dynamic contrast-enhanced CT (CECT) and screened. According to the deep learning multilayer perceptron (MLP), 15 optimal features were optimized to establish the radiomic feature score (Rad-score), and LASSO regression was used to screen out clinically independent predictors. Based on logistic regression, the Rad-score and clinically independent predictors were integrated to build the clinical radiomics model and visualized as a nomogram and independently verified in the testing set. The performance and clinical applicability of hybrid model in identifying MSI status were evaluated by the area under the receiver operating characteristic (AUC) curve, calibration curve, and decision curve (DCA).

**Results:**

The AUCs of the clinical image model in training set and testing set were 0.883 [95% CI: 0.822–0.945] and 0.802 [95% CI: 0.666–0.937], respectively. This hybrid model showed good consistency in the calibration curve and clinical applicability in the DCA curve, respectively.

**Conclusions:**

Using preoperative imaging and clinical information, we developed a deep-learning-based radiomics model for the non-invasive evaluation of MSI in GC patients. This model maybe can potentially support clinical treatment decision making for GC patients.

**Graphical abstract:**

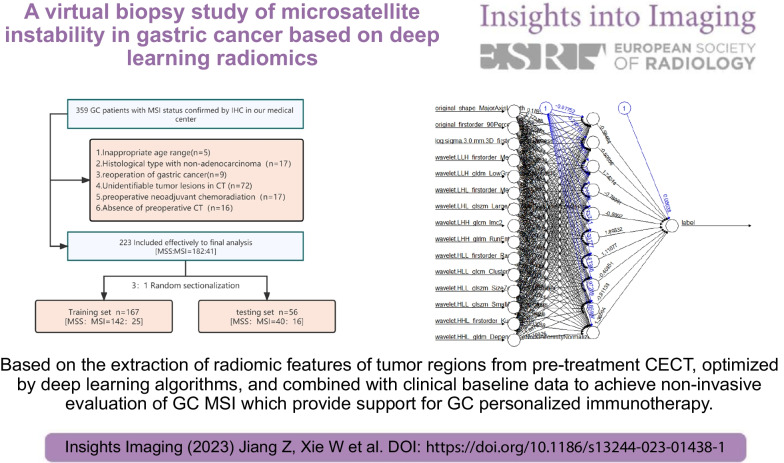

**Supplementary Information:**

The online version contains supplementary material available at 10.1186/s13244-023-01438-1.

## Key points

- MSI is an important biomarker for immunotherapy in gastric cancer.
- Quantitative radiomics features were closely related to MSI in gastric cancer.
- Combining clinical and radiomics features with deep learning could evaluate MSI noninvasively.

**Supplementary Information:**

The online version contains supplementary material available at 10.1186/s13244-023-01438-1.

## Introduction

Gastric cancer (GC) is a highly heterogeneous malignancy caused by multiple factors and is a global public health problem. The incidence of GC varies according to geographical location and is particularly high in Asia (age-standardized [global] incidence: 32.5 per 100,000 men; 13.2 per 100,000 women) [1, 2]. MSI-positive GC is one of the major molecular subtypes of GC as defined by the Cancer Genome Atlas Group and accounts for 10–22% of all GC patients [3]. The deletion of any of the mismatch gene repair proteins (MLH1, PMS2, MSH2, and MSH6) leads to microsatellite instability (MSI/MSI-H) [4, 5]. In recent years, immune checkpoint inhibitors have shown great potential in the treatment of progressive GC [6]. MSI is an important predictive biomarker for evaluating the effect of anti-programmed cell death-1 (PD-1) immunotherapy in GC patients. Several clinical trials have confirmed that the objective response rate and survival were significantly better in the MSI group than in the microsatellite stabilization (MSS) group in anti-PD-1 immunotherapy for advanced GC [7–9]. However, there is a large individual variation in the efficacy of anti-PD-1 therapy [10], so it is important to select patients who are most likely to benefit. Currently, immunohistochemical staining (IHC) and PCR molecular testing are mainly used to detect MSI expression levels [5, 11, 12]. Given the spatial heterogeneity of MSI expression, a small piece of tissue obtained by invasive biopsy may not be sufficiently representative of the entire tumor region [13], thus affecting the assessment of MMR protein expression. Although universal testing for MSI has been recommended in the NCCN guidelines for patients with GC [14], many patients are not tested due to the invasive, time-consuming and expensive nature of tissue biopsy. Therefore, there is an increasing need to develop a non-invasive method for the holistic assessment of MSI expression in GC.

With the development of computer-aided medicine, accurate assessment of tumor’s pathological features has been achieved by combining radiomics and deep learning to extract and analyze quantitative radiological features, known as 'virtual biopsies,' which provide a reference standard for conventional biopsies [15–17]. Several studies have demonstrated the potential role of deep-learning-based radiomics in predicting lymph node metastasis [18] or response to neoadjuvant chemotherapy [19, 20] in GC, so this study explored the non-invasive assessment of GC biomarkers based on such methods using preoperative computed enhanced tomography (CECT) images and clinical data. It was also visualized as a nomogram to evaluate the potential application as a virtual biopsy tool in clinical auxiliary diagnosis.

## Methods

### Patient selection and clinical variables collection

**Fig. 1:**
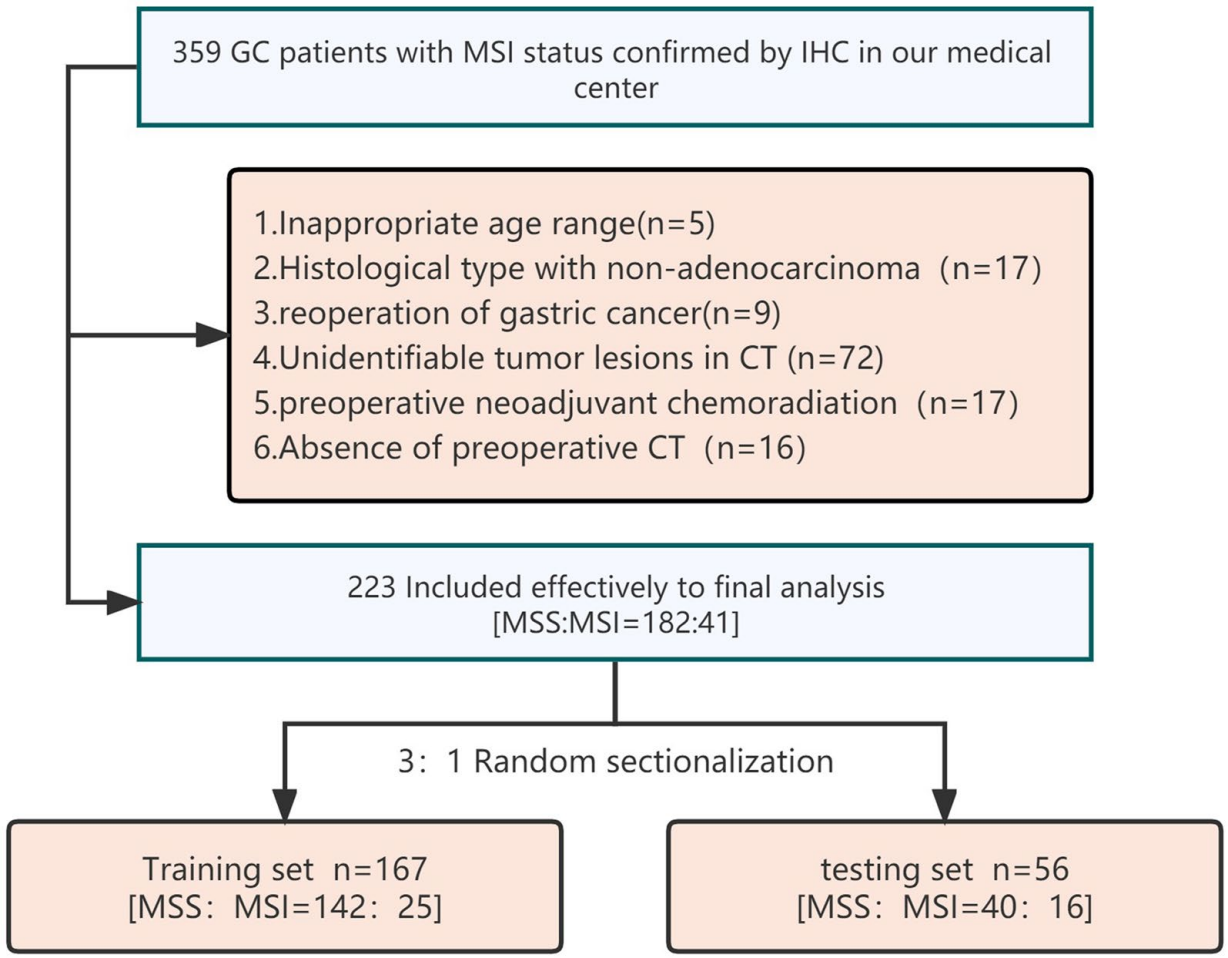
Flowchart of participants recruitment for this study

This study was approved by the ethics committee of our medical center, and the requirement for informed consent was waived due to the retrospective nature of this study. A total of 223 patients were enrolled in this study with MSI confirmed by postoperative IHC of GC by searching the medical database of our hospital from January 2020 to March 2022, including 182 MSS patients and 41 MSI patients. All samples were divided into a training set (*n* = 167) and testing set (*n* = 56) according to the 3:1 random allocation principle. The inclusion criteria were as follows: 1) aged 18–80 years; 2) first gastric cancer surgery; 3) histological type adenocarcinoma; 4) dynamic contrast-enhanced CT of the upper abdomen within 2 weeks before surgery; 5) tumor morphology identified in medical images; and 6) no neoadjuvant chemoradiotherapy performed before surgery. The details of cohort inclusion are shown in Fig. 1.

In this study, we collected baseline clinicopathological data and laboratory parameters measured by venous blood collection within 1 week before surgery, as detailed in Additional file 1: Table 1. According to the clinical application standards of our hospital, the tumor location was reported by professional radiologists after reading the preoperative CECT. According to the eighth edition of the AJCC staging report, the clinical T stage of the tumor was determined.

**Table 1:** Univariate logistic regression analysis of training and testing sets characteristics

	Training set (*n* = 167)			Testing set (*n* = 56)		
Clinical characteristics	MSS/MSI-L (*N* = 142)	MSI/MSI-H (*N* = 25)	*p* value	MSS/MSI-L (*N* = 40)	MSI/MSI-H (*N* = 16)	*p* value
Gender:			**0.015**			0.129
Female	33 (23.2)	12 (48.0)		11 (27.5)	8 (50.0)	
Male	109 (76.8)	13 (52.0)		29 (72.5)	8 (50.0)	
Age (years):	63.29 ± 9.59	68.12 ± 5.95	**0.016**	61.03 ± 10.40	64.25 ± 8.58	0.277
Clinical T stage:			**0.025**			**0.009**
I	2 (1.4)	6 (24.0)		0 (0)	4 (25.0)	
II	21 (14.8)	2 (8.0)		5 (12.5)	1 (6.2)	
III	87 (51.5)	16 (64.0)		21 (52.5)	9 (56.3)	
IV	32 (32.3)	1 (4.0,)		14 (35.0)	2 (12.5)	
Differentiated degree			**0.013**			**0.001**
Well-differentiated	2 (1.4)	1 (4.0)		0 (0)	0 (0)	
Moderately differentiated	42 (29.6)	14 (56.0)		13 (32.5)	13 (81.3)	
Poorly differentiated	98 (69.0)	10 (40.0)		27 (67.5)	3 (18.7)	
Tumor location:			0.765			0.189
Upper-third	22 (15.5)	2 (8.0)		6 (15.0)	0 (0)	
Middle-third	33 (23.2)	6 (24.0)		10 (25.0)	2 (12.5)	
Lower-third	83 (58.5)	16 (64.0)		23 (57.5)	13 (81.3)	
Multiple	4 (2.8)	1 (4.0)		1 (2.5)	1 (6.2)	
Bowman type			0.460			0.365
I	5 (3.8)	2 (8.3)		0 (0)	1 (6.7)	
II	11 (8.3)	1 (4.2)		5 (12.5)	3 (20.0)	
III	114 (85.7)	20 (83.3)		33 (82.5)	11 (73.3)	
IV	3 (2.2)	1 (4.2)		2 (5.0)	0 (0)	
CEA level (ng/mL)			0.658			0.763
Normal	86 (61.4)	16 (66.7)		25 (62.5)	11 (68.8)	
Abnormal	54 (38.6)	8 (33.3)		15 (37.5)	5 (31.3)	
AFP level (ng/mL)			1.000			0.550
Normal	131 (93.6)	23 (95.8)		37 (92.5)	16 (100.0)	
Abnormal	9 (6.4)	1 (4.2)		3 (7.5)	0 (0)	
CA19-9 level (u/mL)			1.000			0.416
Normal	123 (88.5)	21 (91.3)		33 (89.2)	15 (93.8)	
Abnormal	16 (11.5)	3 (8.7)		4 (10.8)	1 (6.2)	
ALB (g/L)	40.10 ± 5.04	40.53 ± 4.82	0.698	40.21 ± 4.70	39.21 ± 5.57	0.501
NE (10^9/L)	3.44 (2.71–4.52)	4.72 (2.68–5.51)	0.061	3.46 (2.52–4.65)	3.67 (2.72–5.10)	0.737
Lym (10^9/L)	1.67 (1.37–2.00)	1.88 (1.07–2.47)	0.474	1.66 (1.37–2.07)	1.50 (1.00–2.50)	0.544
NLR	1.96 (1.54–2.91)	2.33 (1.99–3.19)	0.230	2.07 (1.91–2.83)	1.77 (1.02–3.23)	0.154

All* p* values <0.05 are bolded in Table, which were considered statistically significant in the corresponding correlation analysis

Data for continuous variables are expressed as median (interquartile range) or mean ± standard deviation, and categorical variables are expressed as sample size (%). *AFP* Alpha fetal protein; *ALB* Albumin; *CEA* Carcinoembryonic antigen; *CA19-9* Carbohydrate antigen 19–9; *Lym* Lymphocyte count; *NE* Neutrophil count; *NLR* Neutrophil lymphocyte ratio

### MSI status definition and revaluation

In this study, the MSI status was obtained by immunohistochemistry (IHC), and the immunohistochemical sections were formalin-fixed paraffin embedding with a thickness of 2–3 µm, passed through a standard streptavidin–biotin–peroxidase procedure, and stained by an automatic immunohistochemical staining machine (Leica Bond-Max, Leica Biosystems). Two pathology experts in the field of gastrointestinal tumors (with 8 years and 10 years of work experience, respectively) reanalyzed the expression of four MMR proteins, MLH1, PMS2, MSH2, and MSH6, in IHC sections to characterize MSI. They were unaware of the clinical and pathological information of the samples in advance, and if the results were different, they reached an agreement through consultation. Loss of expression of any MMR protein was defined as defects of mismatch repair and divided into the MSI/MSI-H group; expression of all MMR proteins was defined as professional MMR and divided into the MSS/MSI-L group.

### Protocol of CECT image acquisition

All patients underwent CECT with a 64-slice multislice CT scanner, which covered the entire upper abdomen. The specific parameters of the scanner are detailed in Additional file 1: Table 2. To ensure an empty stomach, all patients fasted for 8 h. Before the examination, they drank more than 800 mL of purified water to fill the stomach cavity. During the examination, the patients were placed in a supine position and asked to hold their breath. A nonionic contrast agent (Iohexol-350 Injection; Starry Pharmaceutical) was pumped into the antecubital vein through an automated high-pressure pump injection system (Medrad Vistron Plus, Bayer Healthcare) at a dose of 1.5 mL/kg, the injection speed was 3 mL/s, and the portal venous phase CECT image was acquired 60 s after the contrast agent was injected. All CT images were reconstructed with an axial thickness of 5 mm. Then, the DICOM format image files were retrieved and exported from the image archiving and communication system and medical imaging workstation and stored for further image segmentation and analysis.

**Table 2:** Selected optimal features in radiomics

	Features	Mean	SD
F1	original_shape_MajorAxisLength	0.344	0.186
F2	original_firstorder_90Percentile	0.337	0.165
F3	log.sigma.3.0.mm.3D_firstorder_Skewness	0.546	0.187
F4	wavelet.LLH_firstorder_Median	0.851	0.115
F5	wavelet.LLH_gldm_LowGrayLevelEmphasis	0.076	0.130
F6	wavelet.LHL_firstorder_Median	0.962	0.078
F7	wavelet.LHL_glszm_LargeAreaLowGrayLevelEmphasis	0.051	0.112
F8	wavelet.LHH_glcm_Imc2	0.299	0.145
F9	wavelet.LHH_glrlm_RunEntropy	0.526	0.130
F10	wavelet.HLL_firstorder_Range	0.198	0.133
F11	wavelet.HLL_glcm_ClusterProminence	0.047	0.116
F12	wavelet.HLL_glszm_SizeZoneNonUniformity	0.187	0.145
F13	wavelet.HLL_glszm_SmallAreaEmphasis	0.527	0.149
F14	wavelet.HHL_firstorder_Kurtosis	0.099	0.151
F15	wavelet.HHL_gldm_DependenceNonUniformityNormalized	0.197	0.152

### Tumor segmentation

**Fig. 2:**
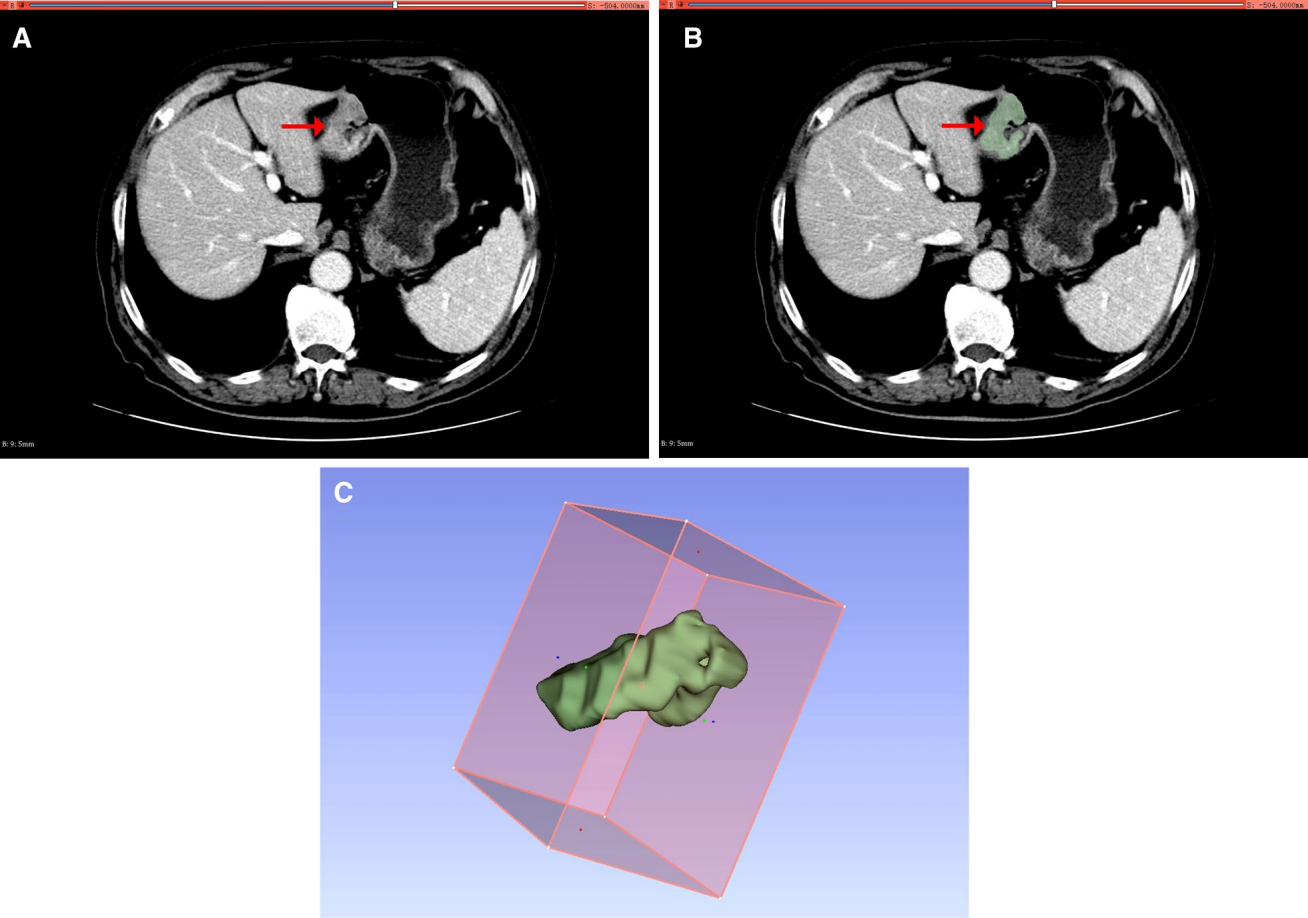
An example of ROI segmentation of CECT images. **A** The portal venous phase scan reveals heterogeneous enhancement of the tumor region (shown by arrow). **B** Semi-automatic segmentation of tumor area (green regions). **C** The full-thickness tumor region was segmented and reconstructed to generate a three-dimensional model of ROI. *CECT* computer-enhanced tomography; *ROI* regions of interest

All CT images were independently reviewed by two radiologists with 5 years (reader 1) and 8 years (reader 2) of experience in gastrointestinal oncology radiology who were blinded to the IHC results. If they disagree on the diagnosis, the final result will be decided by a chief radiologist with more than 20 years of experience in diagnosing abdominal tumors. Using the 3D Slicer software (4.11, www.slicer.org) in reading the CECT image, set to the abdomen window (width: 350 HU; horizontal: 40 HU). Afterward, Reader 1 and Reader 2 utilized the Segmentation Wizard plugin in 3D Slicer to achieve semi-automatic segmentation of tumor boundaries in all axial portal CECT to obtain the region of interest (ROI). After full-slice annotation, a three-dimensional (3D) image was generated to directly reflect the ROI shape, as shown in Fig. 2. During the labeling process, intragastric air, surrounding adipose tissue, areas of tumor necrosis, and perigastric lymph nodes were carefully excluded. Before feature extraction, all images are z-scores normalized separately and resampled at a pixel spacing of 1*1*1 mm.

### Radiomic feature extraction

PyRadiomics (http://www.radiomics.io/) was used to extract radiomics features from all segmented CT images [21]. The extracted feature types included: 1) shape-based (SB); 2) first-order statistics (FOS); 3) gray-level co-occurrence matrix (GLCM); 4) gray-level run-length matrix (GLRLM); 5) gray-level size zone matrix (GLSZM); and 6) gray-level correlation matrix (GLDM), which is consistent with previous studies [22, 23]. To reduce the effect of overfitting of radiomic features on the performance of prediction models, we applied intraclass correlation coefficients (ICCs) to ensure the robustness of radiomic features, and 30 randomly selected CT images from the original reader 1 and reader 2 annotated CT images were used to assess interobserver ICC. After a 4-week interval, reader 1 redrew the ROI of the drawn random sample, extracted the radiomic features with the same process, and calculated the radiomic features of the two repeats of reader 1 to evaluate the intraobserver ICC. Usually, ICC > 0.75 is defined as good consistency, so we discard the features of intragroup and intergroup ICC < 0.75 to ensure robustness.

**Fig. 3:**
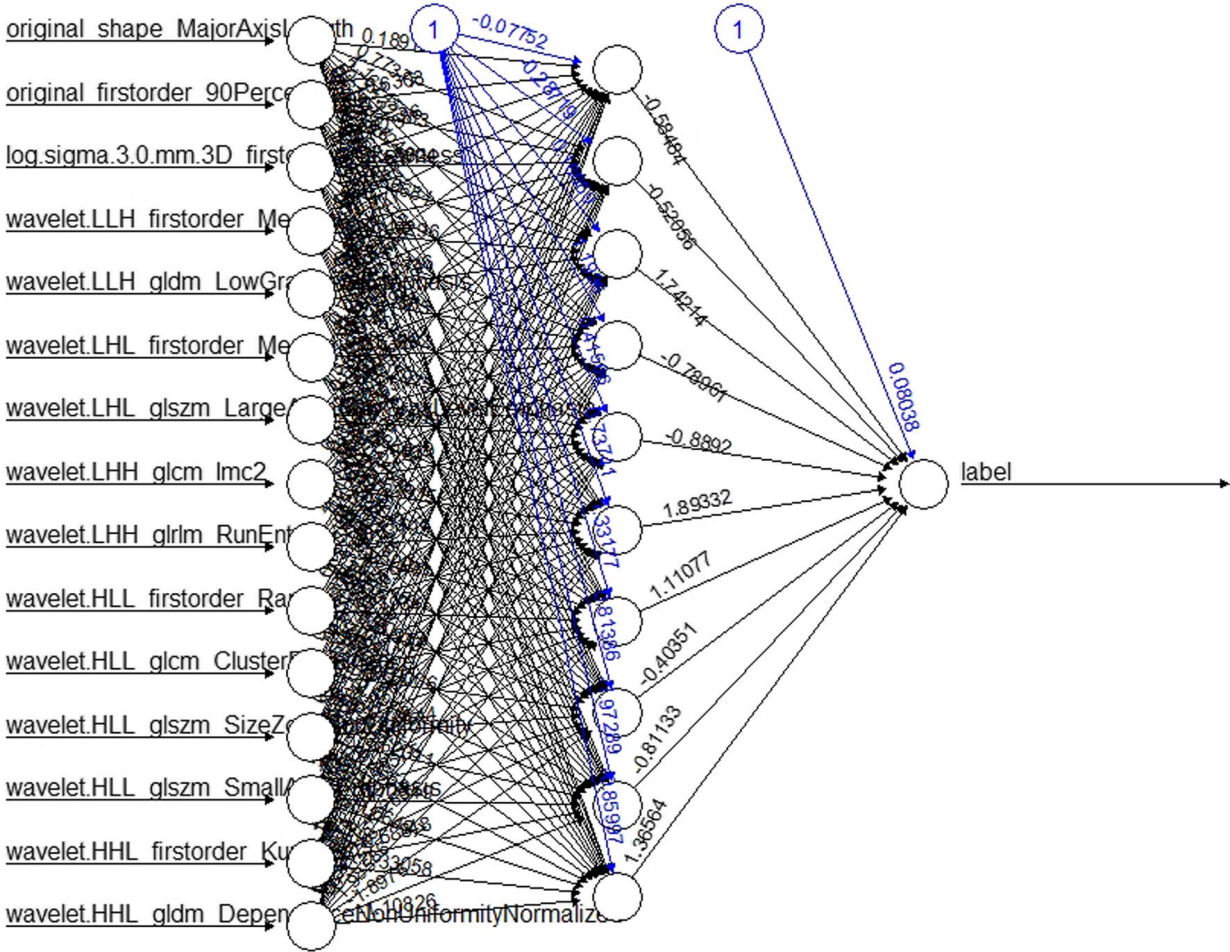
Core radiomics features are quantified using the DL-MLP model to establish a radiomic feature score. *DL-MLP* deep learning multilayer perceptron

To remove redundant features, we employ variance and correlation filters. The specific steps are as follows: If the normalized standard deviation of a feature is less than 0.1, the feature will be discarded because it is invalid; at the same time, the Pearson correlation coefficient of each pair of features is calculated. If the Pearson correlation coefficient between two features is greater than 0.9, the two features are highly similar, excluding one of the two features. All the features were standardized with min–max normalization in both cohorts using the min and max deviation of the training cohort feature data. To make the screened features more predictive, we used a deep learning algorithm, multilayer perceptron (MLP), to quantify the optimal radiomic features and established a radiomic feature score (Rad-score) for each patient (see Fig. 3).

### Development of prediction models and nomogram

To explore whether there is an additional gain in clinical information for predicting MSI status, we performed a univariate logistic regression analysis on clinical data. Then, clinical risk predictors were screened based on LASSO regression, and three independent MSI prediction models (clinical model, radiomics model, and clinical imaging model) were developed using the Rad-score and clinical risk predictors in training set and independently verified in the testing set. Model prediction performance was assessed by the area under the receiver operating characteristic (ROC) curve (AUC), specificity, and sensitivity. Moreover, we visualized the hybrid model as a nomogram using logistic regression to increase the clinical application value. Nomogram performance was evaluated using a calibration curve, and the clinical utility of the nomogram was evaluated by decision curve analysis (DCA) to calculate the maximization of net gain within range thresholds.

### Statistical analysis

IBM SPSS Statistics (26.0; IBM Corp.) was used to conduct a univariate logistic regression analysis, the Chi-square test was used to analyze categorical variables, and the two-sided independent samples t test was used for continuous variables subject to normal distribution. Continuous variables that did not have a normal distribution were analyzed using the Mann‒Whitney U test. A two-sided *p* value of < 0.05 was considered statistically significant. MLP model development, prediction models, nomogram construction, and performance evaluation were all developed through the R (version 3.6.1; http://www.R-project.org) software package.

## Results

### Clinical and pathological characteristics

Univariate logistic regression analysis was performed on clinical and pathological characteristics, and details of the relationship between patient characteristics and MSI status are shown in Table 1. The results showed that there were significant differences between MSI and clinical T stage (*p* value < 0.05) and degree of differentiation (*p* value < 0.05) in the two sets. In the training set, there were significant differences in MSI status in patients of different ages (*p* value = 0.016) and of either sex (*p* value = 0.015). However, tumor location and carcinoembryonic antigen (CEA) level did not show a significant correlation with MSI status in either cohort.

### Radiomics feature selection

**Fig. 4:**
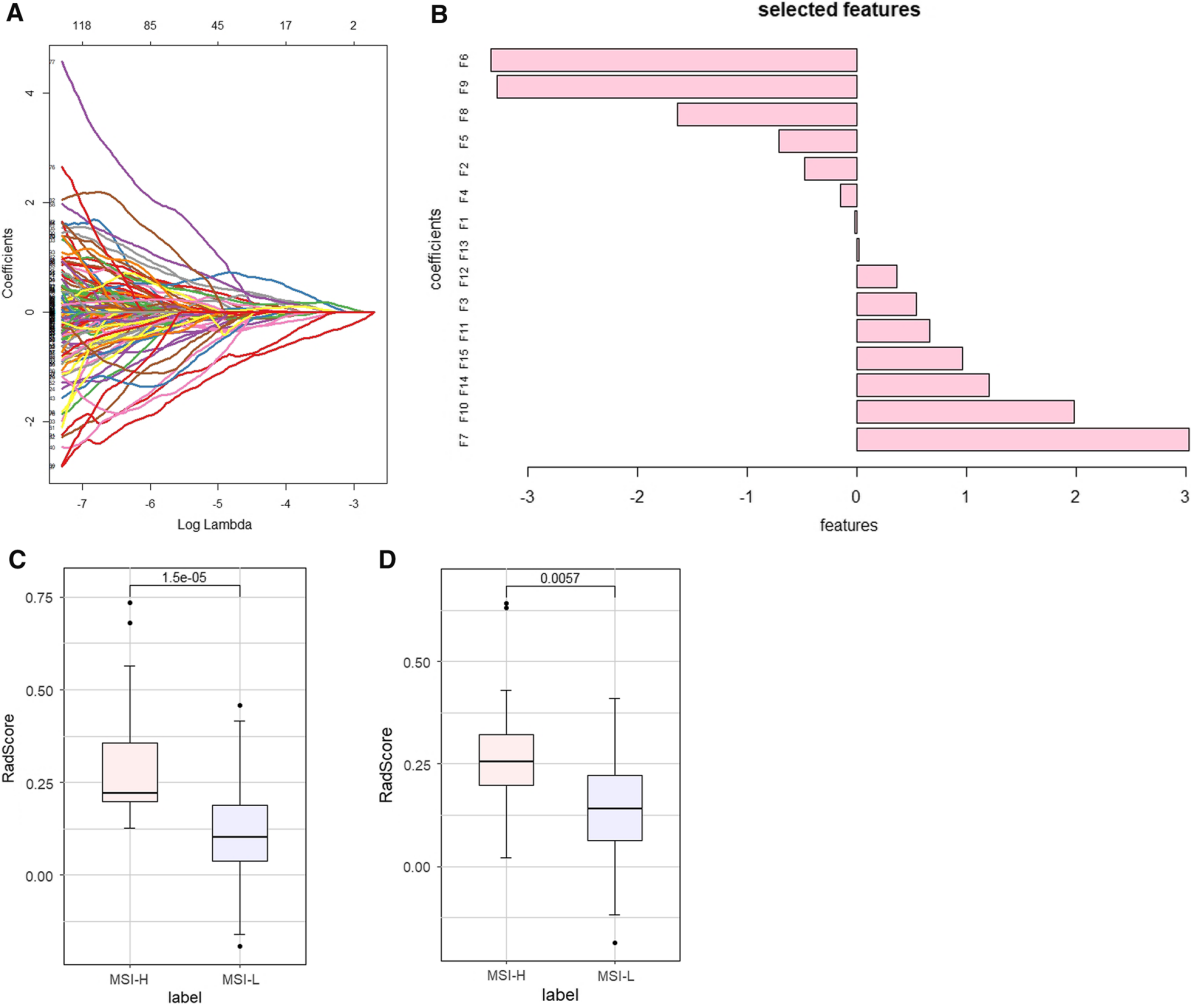
Optimal radiomics features selection and the distribution of Rad-score. **A** The least absolute shrinkage and selection operator (LASSO) binary logistic regression was used to select 15 nonzero features with the highest coefficient. **B** The 15 nonzero coefficients radiomics features subset distribution. The boxplots of Rad-score distribution for the MSI group and MSS group in the training (**C**) and testing (**D**) sets

A total of 982 radiomic features were extracted from each ROI, and 365 features with ICC ≤ 0.75 were excluded after the consistency detection of the features. After that, variance and correlation filters were used to eliminate redundant features, and 197 radiomic features were retained. Finally, using LASSO regression with tenfold cross-validation, 15 optimal radiomic features were screened (Fig. 4A, B), including two original features, one 3D skewness feature, four first-order wavelet features, two GLDM features, three GLSZM features, two GLCM features and one GLRM feature (Table 2).

### Construction of the radiomics signature

**Fig. 5:**
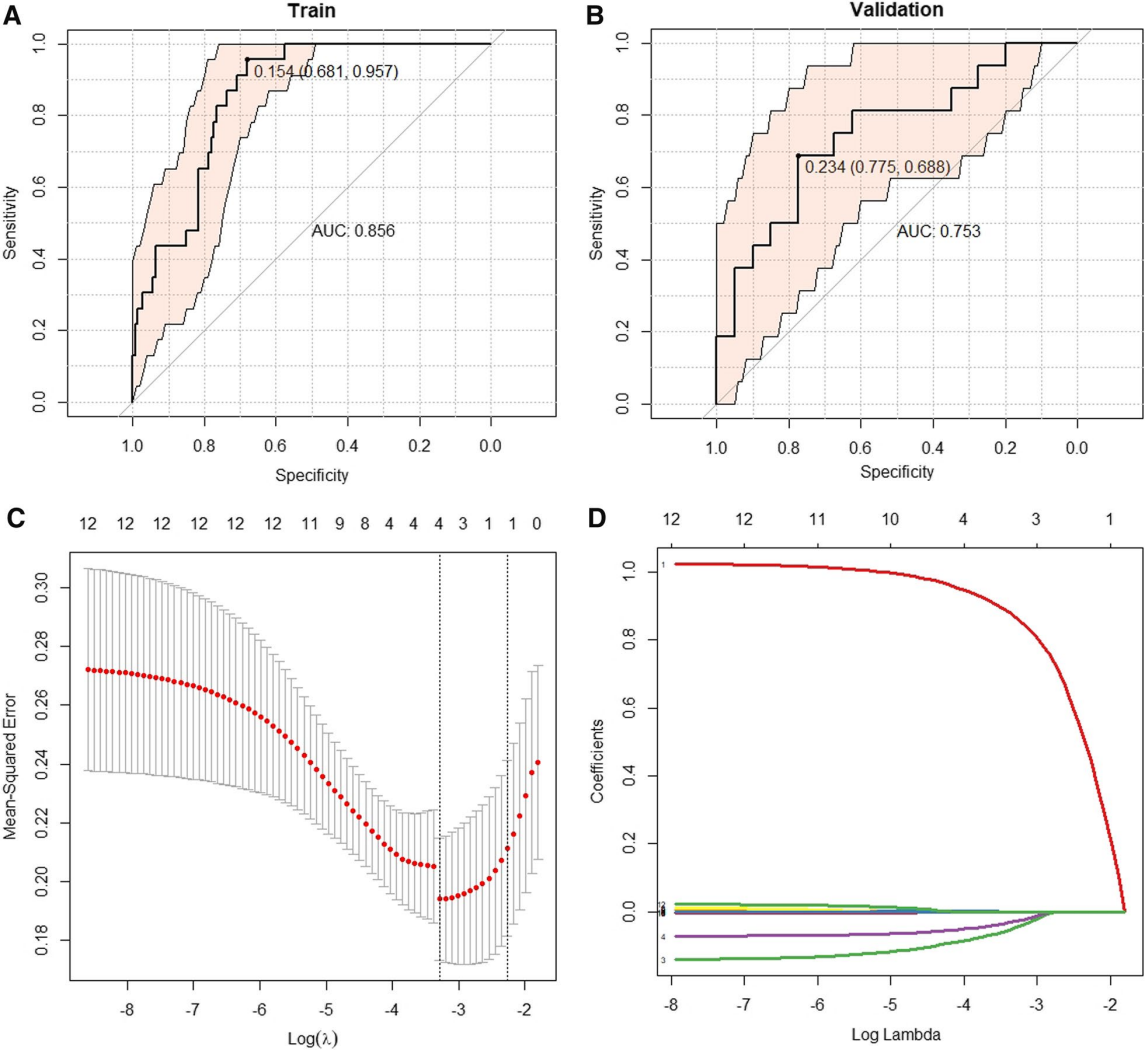
ROC curve of independent Rad-score model and model predictors screening. Independent radiomics model ROC curves of the training set (AUC: 0.856, 95%CI: 0.792–0.919) (**A**) and testing set (AUC: 0.753, 95% CI: 0.606–0.901) (**B**). By the LASSO regression to independent predictors selection. **C** Tuning parameter (lambda, λ) of the LASSO model was selected and optimized by the tenfold cross-validation, and the optimal λ value was obtained by drawing vertical dotted lines. **D** Substituting the optimal λ values into the eigencoefficients, four nonzero coefficient features are obtained. *ROC* operating characteristic curve; *AUC* area under the curve; *95% CI* 95% confidence interval

In the MLP model developed based on a deep learning algorithm, the Rad-score represents quantified radiomic features. We then investigated the distribution of each Rad-score in the two sets. The average Rad-score in the MSI group was significantly higher than that in the MSS group (Fig. 4C, D), which was demonstrated in the testing set. The AUC of the radiomics model based on the independent Rad-score was 0.856 [95% confidence interval (CI): 0.792–0.919] in the training set and 0.753 [95% CI: 0.606–0.901] in the testing set (Fig. 5A, B).

### Evaluation of the models and nomogram

**Fig. 6:**
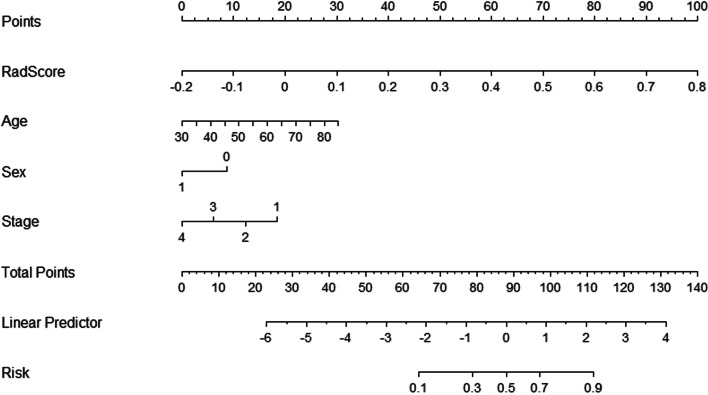
Development of nomogram for predicting MSI status. The nomogram was built based on four independent predictors of the training set, including Rad-score, age, sex, and clinical T stage. In Rad-score, numbers represent scores. In age, the numerical value represents age, increasing by 5 years for each cell. In sex, 0 on behalf of the woman and 1 on behalf of the man. In the stage, the increasing numbers each mean T1, T2, T3, and T4

In univariate analysis, age, sex, clinical T stage, and degree of differentiation were found to be closely related to MSI status, and subsequent LASSO regression analysis identified age, sex, clinical T stage, and Rad-score as independent predictors of MSI (Fig. 5C, D). Ultimately, we constructed three prediction models (clinical model, radiomics model, and clinical image model) based on the above independent predictors and visualized the clinical imaging model as a nomogram (Fig. 6).

**Fig. 7:**
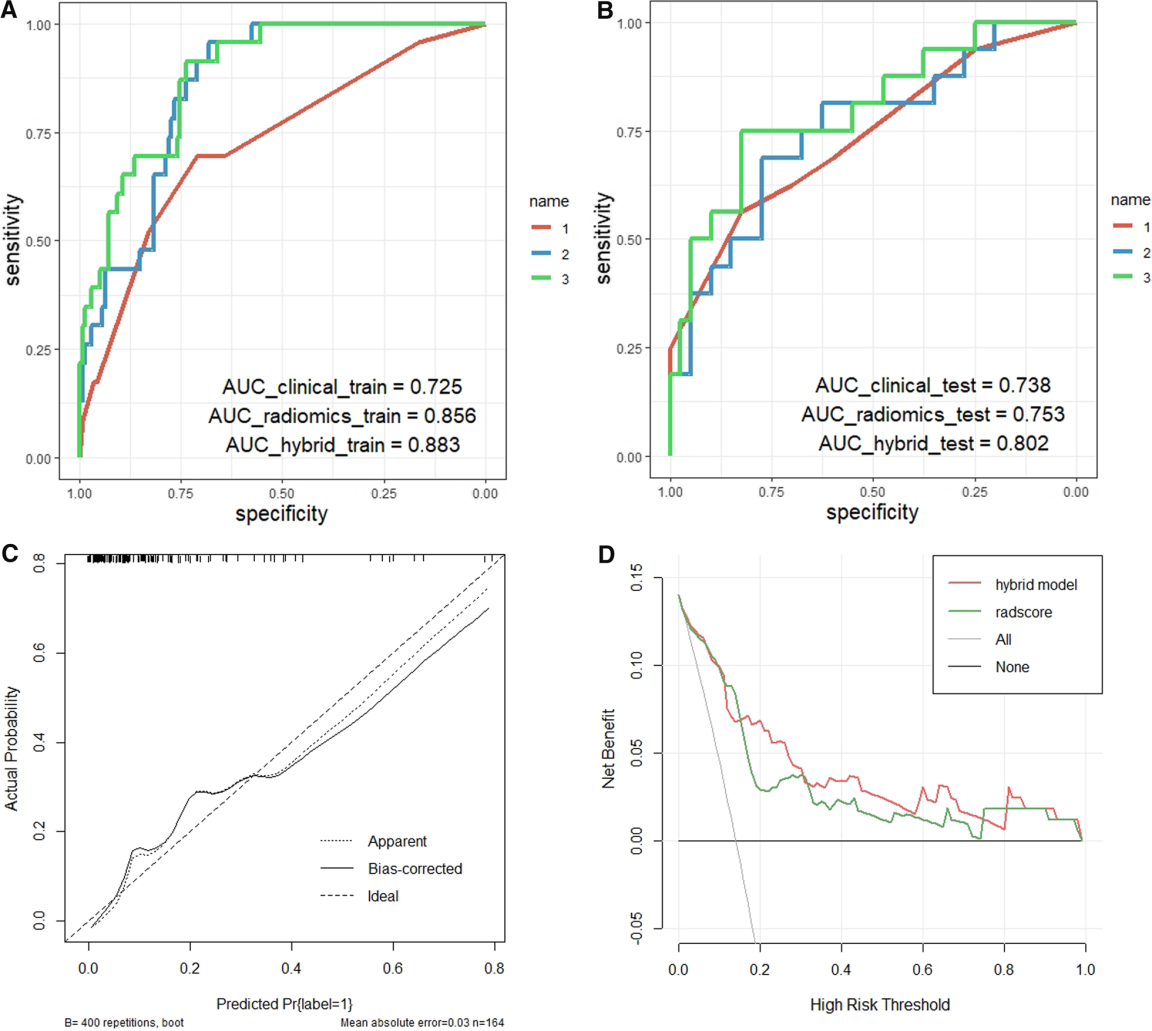
Performance evaluation of prediction models and nomogram. Receiver operating characteristic (ROC) curves comparison of three prediction models in training (**A**) and testing (**B**) sets: Clinical characteristics model (name 1), radiomics features model (name 2), and hybrid model (name 3) of Rad-score combined with the clinical features. As shown in the figure, the hybrid model achieved the highest AUC (0.883 and 0.802) in both sets. **C** The calibration curve shows the calibration between the predicted risk of the MSI state and the observed result of the MSI state in the nomogram model. **D** The DCA of radiomics model and nomogram model. The x-axis represents the risk threshold probability, and the y-axis is the net benefits. The nomogram model showed better clinical net benefits. *AUC* area under the curve

To evaluate the predictive performance of the three models, we plotted the ROC curves and calculated the AUC for comparison (Fig. 7A, B). The AUCs of the clinical models in the training and testing sets were 0.725 [95% CI: 0.611–0.840] and 0.738 [95% CI: 0.586–0.889], respectively. As shown in Fig. 7C, the calibration curve for the nomogram shows good agreement between the observed and predicted results. To assess the clinical applicability of the nomogram, we performed a clinical decision curve analysis on the nomogram (Fig. 7D). At a threshold probability of 20%–70%, the nomogram model showed a greater net benefit than treating all patients or no treatment compared to the stand-alone radiomics model and at the 0%–20% and 70%–100% ranges with similar net gains.

Compared with separate clinical and radiomics models, the clinical image model combining preoperative clinical features and Rad-score showed better predictive performance in classifying MSI and MSS status; AUCs of 0.883 [95% CI: 0.822–0.945] in training set and 0.802 [95% CI: 0.666–0.937] in testing set were achieved. The prediction performance details of the three prediction models are shown in Table 3.

**Table 3:** Comparison of the prediction performance of three models for MSI status

Models	AUC (95%CI)	Sensitivity (95%CI)	Specificity (95%CI)
Clinical model			
Training (*n* = 167)	0.725 (0.611–0.840)	0.683 (0.478–0.870)	0.775 (0.404–0.851)
Testing (*n* = 56)	0.738 (0.586–0.889)	0.553 (0.300–0.812)	0.812 (0.454–0.960)
Radiomics model			
Training (*n* = 167)	0.856 (0.792–0.919)	0.957 (0.782–1.000)	0.681 (0.518–0.794)
Testing (*n* = 56)	0.753 (0.606–0.901)	0.625 (0.312–0.875)	0.750 (0.325–0.925)
Hybrid model			
Training (*n* = 167)	0.883 (0.822–0.945)	0.870 (0.565–1.000)	0.723 (0.539–0.823)
Testing (*n* = 56)	0.802 (0.666–0.937)	0.688 (0.375–0.938)	0.775 (0.370–0.950)

*AUC* Area under the curve; *95% CI* 95% confidence interval

## Discussion

We used clinical information and pretreatment CECT images of 223 GC patients from our medical center to develop a virtual biopsy model supported by deep learning algorithms. To the best of our knowledge, we are the first to apply deep learning algorithms to the optimization of radiomics feature building in the GC MSI radiomics. Clinical imaging models supported by MLP effectively improve the ability to identify MSI/MSI-H in GC patients (AUC: training set 0.883, testing set 0.802). Accurate MSI identification is critical for individualized systemic treatment of GC patients, which can benefit patients receiving anti-PD-1 immunotherapy and improve GC patient survival [9, 24, 25].

All valid samples in a unit period were covered in this study, avoiding selection bias as much as possible. The incidence of MSI in GC patients in the study was 18.4%, which is consistent with the current Global epidemiology on GC-related MSI (10–22%) [3, 5, 26]. LASSO regression analysis of clinical baseline data found that age, sex, and clinical T stage were independent clinical risk factors closely related to MSI expression in GC patients. Considering the relationship between advanced age [27–30] and female sex [28, 30], GC patients and MSI are consistent with previous related studies. Current evidence suggests that the MSI-H phenotype in patients with sporadic GC is closely associated with hypermethylation of the promoter CpG island causing silencing of the hMLH1 gene, which leads to progressive loss of MLH1 protein expression [31–33]. Similar findings were found in our recruited patients, with more than 83% (*n* = 35) of MSI-expressing patients having a deletion in the expression of the mismatch repair protein MLH1. Interestingly, researchers such as Nakajima et al. [34] and Kim et al. [35] found that the methylation of the mismatch repair gene hMLH1 was age dependent, and its incidence was positively correlated with age. hMLH1 methylation is more common in elderly gastric cancer patients, which seems to explain why MSI/MSI-H was more common in older GC patients in our study. Compared with MSS/MSI-L, GC with the MSI/MSI-H phenotype is less aggressive, representing a better prognosis in the early stage of GC [27, 36]. At the molecular level, recent studies have found that changes in the genetic and epigenetic characteristics of the GC genome often occur in the early stages of the tumor [37, 38], which supports that hMLH1 promoter methylation-induced MSI-H is more likely to be seen in the early stage (TNM stages I–II) presumed in GC patients, thus defining MSI as an early molecular event of GC. This is also confirmed in the reports of Polom et al. [30] and Jahng et al. [39]. However, we did not observe the previously demonstrated significant relationship between tumor location [29, 30, 36] and MSI, which may be related to differences in the patient population or regional prevalence of our recruitment. Taken together, these potentially characteristic clinical factors reflect higher histological heterogeneity in MSI tumors.

The development of radiomics technology makes it possible to capture the heterogeneity of tumor molecules in clinical images, which can provide objective and quantitative support for cancer molecular biological detection and personalized treatment [40]. We finally selected 15 radiomics features (including 1 first-order feature, 1 shape-based feature, and 13 filtered features) from the filtered and unfiltered images to build the model. The first-order feature describes the distribution of voxel intensities in CT images, and the shape-based feature is a description of the two-dimensional tumor regions’ size and shape. The filtered features are first-order statistical features and texture features extracted from the filtered images and reconstructed by transforming the Laplacian of the Gaussian spatial bandpass filter or wavelet filter, where the texture features include the gray-level co-occurrence matrix (GLCM), gray-level run-length matrix (GLRLM), gray-level size zone matrix (GLSZM), and gray-level dependence matrix (GLDM). With these two different filtering strategies, the specific structure of the original image is enhanced. These features reflect the differences in spatial morphology, pixel intensity, and texture of tumor regions and may represent spatial and temporal heterogeneity in tumor tissue and characteristic biological phenotypes [23, 41]. This may explain the ability of the Rad-score established in this study to exhibit differentiated GC MSI in both cohorts. Additionally, accurate and efficient tumor region segmentation methods are important to ensure the quality of quantitative image features. It has been confirmed that 3D Slicer has better segmentation algorithms and higher segmentation accuracy than other image segmentation software while being more accessible as free open-source software [42]. Moreover, the radiomics features extracted after image segmentation using 3D Slicer have higher robustness and are, therefore, recommended for use in high-throughput data mining efforts for medical oncology imaging [43].

The exploration of radiomics in the field of GC biomarkers has started. Li et al. [44] used radiomics to construct a predictive model for detecting GC HER-2 expression, with an AUC of 0.799 [95% CI: 0.704 − 0.894]. Based on the information of 189 patients, Liang et al. [45] first constructed a predictive model based on logistic regression analysis to explore the feasibility of predicting GC-related MSI status, and the AUC was 0.8228 [95% CI: 0.7355–0.9101]. However, traditional radiomic method brings challenges in image segmentation, standardization, acquisition, and reconstruction. As an emerging means of quantitative image analysis, deep learning can optimize such limitations and improve the accuracy and reliability of prediction models [16, 46, 47]. The combination of deep learning and radiomics has shown promising results [18–20, 48]. In this study, we first attempted to apply deep learning algorithms to the calculation and reconstruction of GC-related MSI radiomic features. Compared with previous [45] research, our results were encouraging and obtained a higher AUC (0.883 and 0.802). This may be attributed in the optimization of radiomic features by deep learning algorithm, the increase in sample size, and the level of image segmentation in our study. Additionally, we tried to add pathological features to the combined model, but the final result did not significantly improve the model's predictive performance, reflecting the independent value of pretreatment radiomics in predicting MSI status in GC patients. To increase clinical practicability, we visualized the clinical image model into a nomogram based on logistic regression to generate the prediction probability of MSI so that it could be used as a virtual biopsy tool to support clinical medical decision making.

It should be noted that our study also has limitations. First, this is a single-center retrospective study, and inevitably, there is a patient selection bias. Although we tried to include a relatively more number of cases (*n* = 223), the study sample is still small, considering the high incidence of gastric cancer in Asia, which may affect the generalizability of the model. Second, due to the inherent black box property of machine learning, the process between model data input and output is difficult to interpret, and this lack of transparency has implications for clinical practice, so we plan to apply more transparent and interpretable medical algorithms in future [49, 50]. Finally, we also noted the place of dual-energy CT (DECT) in radiomics, and in future, we plan to collect cases in DECT centers to study the benefit of DECT in GC MSI virtual biopsy studies.

## Supplementary Information

**Additional file 1**. Clinical baseline data indicators and CT scanner parameters.

## Data Availability

The datasets analyzed during the current study are available from the corresponding author on reasonable request.

## Abbreviations

95% CI: 95% Confidence interval
AUC: Area under the curve/area under the receiver operating characteristic
CA19-9: Carbohydrate antigen 19–9
CEA: Carcinoembryonic antigen
CECT: Contrast-enhanced CT
DCA: Decision curve analysis
DECT: Dual-energy CT
GC: Gastric cancer
ICCs: Intraclass correlation coefficients
IHC: Immunohistochemical staining/immunohistochemistry
LASSO: Least absolute shrinkage and selection operator
MLP: Multilayer perceptron
MMR: Mismatch repair
MSI: Microsatellite instability
MSS: Microsatellite stability
PD-1: Programmed cell death-1
Rad-score: Radiomic score
ROC: Operating characteristic curve
ROI: Regions of interest

## Radiomics feature types

FOS: First-order statistics
GLCM: Gray-level co-occurrence matrix
GLDM: Gray-level dependence matrix
GLRLM: Gray-level run-length matrix
GLSZM: Gray-level size zone matrix
SB: Shape-based
