# Supplementary material for: A virtual biopsy study of microsatellite instability in gastric cancer based on deep learning radiomics
Source: Insights Imaging. 2023 Jun 7;14:104. doi: 10.1186/s13244-023-01438-1 (PMC10247640; doi:10.1186/s13244-023-01438-1)
Supplement: Supplementary file 1 — Additional file 1. Clinical baseline data indicators and CT scanner parameters. [file 13244_2023_1438_MOESM1_ESM.pdf]

A virtual biopsy study of microsatellite instability in gastric cancer based on deep learning radiomics

Supplementary Table 1. Clinical baseline data indicators.

**Baseline clinicopathological data**	age	gender	clinical T stage	tumor location	Bowman type	degree of differentiation	MSI status
	**oncological markers**			**nutritional inflammatory markers**			
**Laboratory testing indicators (thresholds)**	CEA (3.4ng/ml)	AFP (7.02ng/ml)	CA-199 (39u/ml)	Albumin (35g/l)	neutrophil counts (3.2×10^9)	Lymphocyte count (0.6×10^9)	Neutrophil lymphocyte ratio

Supplementary Table 2. CT scanner parameters.

**Scanner**	Tube voltage (kV)	Tube current (mAs)	matrix	Pitch (mm)	Rotation time(s)	section thickness (mm)	Detector collimation (mm)
64-slice Multislice CT Scanner (GECT 670; GE medical systems)	120	220	512x512	1.375	0.5	5.00	64x0.625

CT, computed tomography.
